# Analysis of the Antioxidant Mechanism of *Tamarix ramosissima* Roots under NaCl Stress Based on Physiology, Transcriptomic and Metabolomic

**DOI:** 10.3390/antiox11122362

**Published:** 2022-11-28

**Authors:** Yahui Chen, Haijia Li, Shiyang Zhang, Shanfeng Du, Guangyu Wang, Jinchi Zhang, Jiang Jiang

**Affiliations:** 1 Collaborative Innovation Center of Sustainable Forestry in Southern China of Jiangsu Province, Nanjing Forestry University, Nanjing 210037, China; 2 Department of Forest Resources Management and Faculty of Science, University of British Columbia, Vancouver, BC V6T 1Z4, Canada

**Keywords:** NaCl stress, antioxidant mechanism, transcriptome, metabolome, NaCl toxicity

## Abstract

There is a serious problem with soil salinization that affects the growth and development of plants. *Tamarix ramosissima* Ledeb (*T*. *ramosissima*), as a halophyte, is widely used for afforestation in salinized soils. At present, there are few reports on the antioxidant mechanism of *T*. *ramosissima* under NaCl stress. In this study, we learned about the superoxide dismutase (SOD), peroxidase (POD), and catalase (CAT) activities, and hydrogen peroxide (H_2_O_2_) and malondialdehyde (MDA) content changes in *T. ramosissima*. We also mined the relevant metabolic pathways in the antioxidant mechanism, candidate key genes, and their related differential metabolites and verified them using quantitative real-time PCR (qRT-PCR). The results show that the SOD, POD, and CAT activities, and the H_2_O_2_ and MDA content reached the highest values in the roots of *T. ramosissima*. Simultaneously, 92 differentially expressed genes (DEGs) related to antioxidant enzyme activities changed during 48 and 168 h of NaCl stress, and these DEGs were mainly upregulated in 168 h. Based on the association analysis of transcriptomic and metabolomic data, we found *Unigene0089358* and *Unigene0007782* as genes related to key enzymes in the flavonoid biosynthesis pathway. They were located in the upstream positive regulation at 48 and 168 h under NaCl stress, and their respective related metabolites (phloretin and pinocembrin) were involved in resistance to NaCl stress, and they were significantly correlated with their respective metabolites. In conclusion, at 48 and 168 h under NaCl stress, the roots of *T. ramosissima* resist NaCl stress by enhancing enzymatic and nonenzymatic antioxidant mechanisms, scavenging ROS generated by high-salt stress, alleviating NaCl toxicity, and maintaining the growth of *T. ramosissima*. This study provides genetic resources and a scientific theoretical basis for further breeding of salt-tolerant *Tamarix* plants and the molecular mechanism of antioxidants to alleviate NaCl toxicity.

## 1. Introduction

There are a number of factors that influence plant growth, including soil salinization. Currently, about 8 × 109 km^2^ of the land in the world is affected by salinization [1]. There are about 3.6 × 108 km^2^ of salinized soil in China, accounting for about 4.9% of the country’s usable land area [2]. Soil salinization is mainly characterized by excess Na^+^, Mg^2+^, Ca^2+^, CO_3_^2−^, HCO^3−^, Cl^−^, and SO_4_^2−^, especially Na^+^ and Cl^−^ [3].

Salt stress is one of the major environmental factors affecting plant growth and development [4]. In particular, an elevated salt level can lead plants to ion imbalance and osmotic stress, which in glycophitic plants negatively affect their growth and development and eventually lead to serious damage [5,6]. Meanwhile, water shortages caused by salt stress reduces plant stomatal conductance and photosynthetic activity, thereby accelerating the accumulation of reactive oxygen species (ROS) [7]. Salt stress can also cause metabolic imbalance and excessive ROS accumulation in plants, causing oxidative damage and affecting the growth and development of plants as a result of ion imbalance and osmotic stress [8]. When a plant is subjected to salt stress, ROS is produced in many of its organelles and cells. Plants scavenge accumulated ROS by activating an adaptative response [9,10]. Notably, plants have evolved enzymatic and nonenzymatic antioxidant defense systems to improve tolerance to salt stress over a long period of time [11]. The enzymatic system mainly includes enzyme systems, including superoxide dismutase (SOD), catalase (CAT), guaiacol peroxidase (POD), ascorbic acid peroxidase (APX), glutathione peroxidase (GPX), glutathione reductase (GR), glutathione S-transferase (GST), monodehydroascorbate reductase (MDHAR), and dehydroascorbate reductase (DHAR) [12,13]. Under salt stress, the increased activity of these antioxidant enzymes can generally be considered to enhance plant salt tolerance [14]. Nonenzymatic systems include ascorbic acid (AsA), glutathione (GSH), α-tocopherol, phenolic compounds (PhOH), flavonoids, alkaloids, and nonprotein amino acids [15,16]. Enzymatic and nonenzymatic antioxidants produced by plant cells can help eliminate ROS, reduce its damage to plant cells, and improve the antioxidant capacity of plants [17,18]. Therefore, enzymatic and nonenzymatic antioxidants play an important role in scavenging excess ROS accumulation [19], which is essential for maintaining normal plant growth and development [20]. Nonenzymatic antioxidants such as flavonoids can interact with antioxidant enzymes such as SOD, CAT, APX, GPX, GST, and GR in enzymatic antioxidants to prevent the overproduction of ROS [16].

The flavonoid biosynthesis pathway is a plant biology secondary metabolic pathway with the most research [21]. Flavonoids, as plant antioxidants, protect plants from damage resulting from abiotic stresses [22]. Flavonoids, as important secondary metabolites of plants, play an important role in the tolerance response of plants to salt stress and the elimination of hydrogen peroxide (H_2_O_2_) and ROS [18,23]. Flavonoids generally include chalcones, stilbenes, aurones, flavanones, flavones, isoflavones, phlobaphenes, dihydroflavonols, flavonols, leucoanthocyanidins, proanthocyanidins, and anthocyanins [24,25]. The results of Qin et al. showed that the more flavonoids accumulated in plants, the more resistant they were to the damage caused by high salt stress [26].

The root system of plants is the first organ to feel the stress signal under salt stress, and it is also the most directly damaged part [27,28,29]. Under normal growth conditions, the root system can absorb and transport water and nutrients to the aerial parts of the plant to maintain cellular homeostasis. However, under salt stress, changes in the root phenotype and cellular structure disrupt this balance [30,31], and salt stress can inhibit root respiration, affect the normal metabolism of roots, and lead to functional disorders such as root absorption, water, and nutrient transport [32]. However, halophytes are salt-tolerant plants that can achieve growth in high salt concentrations and are adapted to saline–alkali environments [33]. Flowers et al. found that halophytes can complete several life-cycle alternations at concentrations above 200 mM of NaCl [34]. In addition, the *Tamarix* plant as a halophyte [35] contains polyphenols such as flavonoids, tannins, and phenolic acids [36]. The *Tamarix* plant possesses good economic [37,38,39], medicinal [40,41], and ecological values [42], and it is an especially valuable herb in China. It had been used by Chinese doctors for hundreds of years as an herb medicine. Several diseases as well as detoxification can be treated with *Tamarix* plants, according to the Compendium of Materia Medica. Numerous recent studies have been conducted using the *Tamarix* plant extract that support the medicinal benefits of *Tamarix* plants. In addition to their antioxidant, anti-inflammatory, and anticancer properties, *Tamarix* plants are rich in flavonoids and phenols. *Tamarix* plants are widely used in the treatment of diabetes, rheumatoid arthritis, Alzheimer’s disease, and cancer, and in liver protection [14]. *Tamarix ramosissima* Ledeb (*T*. *ramosissima*), a typical representative of *T. ramosissima* plants, normally grows at concentrations below 100 mM of NaCl but inhibits growth at concentrations above 200 mM of NaCl [43].

In this study, the roots of *T. ramosissima* were subjected to 200 mM of NaCl stress. We combined physiological, transcriptomic, and metabolomic data to understand the role of antioxidants in response to NaCl stress in the roots of *T. ramosissima* and observed the changes in their antioxidant mechanisms in anticipation of uncovering key metabolic pathways, candidate genes, and metabolites. Our aim was to provide a scientific theoretical basis and genetic resources for the study of salt toxicity mitigation effects on *Tamarix* plants under NaCl stress.

## 2. Materials and Methods

### 2.1. Plant Material Selection

Five-month-old *T. ramosissima* cuttings with similar growth were selected and transferred them to a 24-hole hydroponic box (40 cm × 30 cm × 16 cm) filled with 1/2 Hoagland nutrient solution. The culture medium was replaced every 3 days and placed in a greenhouse at a temperature of 26 ± 2 °C and relative humidity of 40 to 55%. The experiment was carried out in the key laboratory of the School of Forestry, Nanjing Forestry University.

### 2.2. Experimental Seedling Treatment

The control and treatment groups were set up in the experiment, with 8 plants in each group, and the experiment was repeated three times in total. The control group was cultured in 1/2 Hoagland nutrient solution, and the treatment group was cultured in 1/2 Hoagland nutrient solution supplemented with 200 mM NaCl. The culture solution was changed every 3 days. Then, root samples at 0, 48, and 168 h were collected for treatment, immediately placed in liquid nitrogen, and then transferred to a −80 °C refrigerator for future use.

### 2.3. Determination of Physiological Indexes

We selected 8 *T*. *ramosissima* seedlings under 200 mM NaCl stress at 0, 48, and 168 h after treatment, and each treatment had 3 biological replicates. We sampled the root tissue to determine the SOD, POD, and CAT activities [43], and the H_2_O_2_ [44] and MDA content [45] of *T*. *ramosissima* under different treatments.

### 2.4. Transcriptome Sequencing and Differentially Expressed Gene Screening

We sent the samples of *T*. *ramosissima* root after experimental treatment to GENE Denovo biotechnology company (GENE Denovo, Guangzhou, China) for high-throughput transcriptome sequencing. We extracted total RNA using the Omega kit (Beinuo Bio, Shanghai, China) from Omega Bio-Tek. RNA quality was determined using RNase free agarose gel electrophoresis on an Agilent 2100 Bioanalyzer (Agilent Technologies, Palo Alto, CA, USA). Using an Epicentre Ribo-Zero^TM^ magnetic kit (Madison, WI, USA), mRNA from eukaryotic cells was enriched with Oligo(dT) beads, and mRNA from prokaryotic cells was enriched by removing rRNA. A fragmentation buffer was used to fragment the enriched mRNA, and random primers were used to reverse transcript the cDNA. With the help of DNA polymerase I, RNase H, dNTP, and buffer, second-strand cDNA was synthesized. A QiaQuick PCR extraction kit (Qiagen, Venlo, The Netherlands) was used to purify cDNA fragments, end repair them, add poly(A), and ligate them to Illumina sequencing adapters. Using Illumina HiSeq^TM^ 4000 by Gene Denovo Biotechnology Company (Guangzhou, China), ligation products were size-selected using agarose gel electrophoresis, and the PCR was amplified and sequenced.

Then, we submitted the raw sequencing data obtained by transcriptome sequencing to the Short Reads Archive (SRA) database of the National Center for Biotechnology Information (NCBI), SRP number SRP356215. Finally, we referred to the method of Chen et al. to screen the differentially expressed genes (DEGs) from the sequencing data [46], and the DEGs were obtained for Gene Ontology (GO) [47] and Kyoto Encyclopedia of Genes and Genomes (KEGG) [48] enrichment analysis.

### 2.5. Metabolic Extraction, Detection, and Differential Metabolic Screening

We sent the processed samples of *T*. *ramosissima* root after experimental treatment to GENE Denovo biotechnology company (GENE Denovo, Guangzhou, China) for metabolite extraction and detection and then analyzed via liquid chromatography–mass spectrometry (LC–MS) [49]. We referred to the method of Chen et al. for differential metabolite screening and *p*-value testing of sequencing data [46]. At the same time, we also identified the pathways annotated by differential metabolites in the KEGG database (www.kegg.jp/kegg/pathway.html, accessed on 22 January 2021).

### 2.6. Quantitative Real-Time PCR (qRT-PCR) Validation of Candidate Key Genes

Eight candidate key genes were randomly selected to verify the accuracy of RNA-Seq results. We designed primers for candidate key genes and performed qRT-PCR detection (Supplementary Table S1), which was verified by referring to the method of Chen et al. [50]. Each gene was biologically replicated 3 times, with *Tubulin* as the internal reference gene, and the relative expression was calculated using the 2^−ΔΔCt^ method [51].

### 2.7. Experiment Processing

In this study, we used Excel (Microsoft, Washington, DC, USA) to process all the data, SPSS 26.0 (IBM, Chicago, IL, USA) to perform significant analysis, Origin 2018 software (OriginLab Corporation, Northampton, MA, USA) to make the graph, and MEGA 11 software (MEGA Software, State College, PA, USA) to create phylogenetic trees. Then, we used ANOVA for significance testing, transcriptome sequencing, and metabolite detection which were repeated 3 times technically and 3 times biologically.

## 3. Results

### 3.1. Analysis of Main Antioxidant Enzyme Activities, H_2_O_2_ Content, and MDA Content of T. ramosissima Roots under NaCl Stress

**Figure 1:**
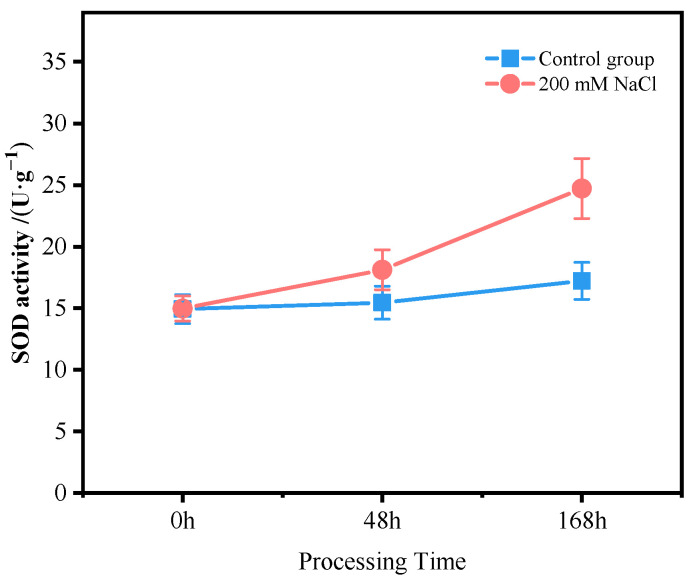

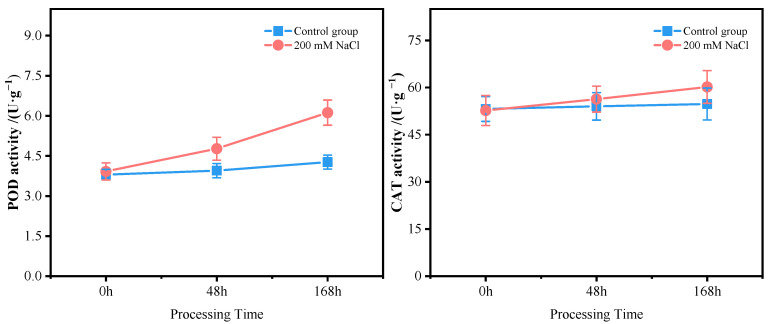
Change in antioxidative enzyme activity of *T. ramosissima* roots under NaCl stress. (Changes in SOD, POD, and CAT activities in the roots of *T. ramosissima* at 0, 48, and 168 h under NaCl stress).

**Figure 2:**
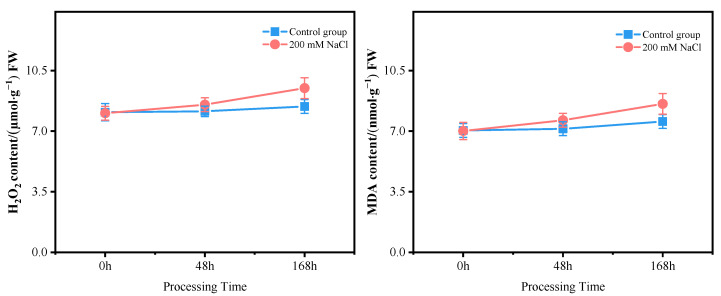
Change in H_2_O_2_ and MDA content of *T. ramosissima* roots under NaCl stress. (Changes in H_2_O_2_ and MDA content in roots of *T*. *ramosissima* under different treatments at 0, 48, and 168 h).

The roots of *T*. *ramosissima* were treated with 200 mM of NaCl stress for 0, 48 and 168 h, and the SOD, POD, and CAT activities increased with time (Figure 1). It is worth noting that the SOD, POD, and CAT activities all reached the highest at 168 h under 200 mM of NaCl stress. The content of H_2_O_2_ and MDA increased with time in the roots of *T*. *ramosissima* treated with 200 mM of NaCl stress for 0, 48 and 168 h (Figure 2). In particular, at 168 h under 200 mM of NaCl stress, the content of H_2_O_2_ and MDA both reached the maximum.

In conclusion, the SOD, POD, and CAT activities, and the H_2_O_2_ and MDA content in the roots of *T*. *ramosissima* within 168 h were induced by NaCl to increase to different degrees.

### 3.2. Analysis of Differentially Expressed Genes Related to Antioxidant Enzyme Activities in the Roots of T. ramosissima under NaCl Stress

DEGs were screened with FDR < 0.05, corrected *p* < 0.05, and |log2 FC| > 1 according to the transcriptional data of *T*. *ramosissima* treated with NaCl stress at 0, 48, and 168 h.

Under NaCl stress for 48 and 168 h, there was the expression of 92 SOD, POD, CAT, APX, GPX, GST, and GR activity-related DGEs in the roots of *T*. *ramosissima* (Supplementary Figure S1). In the comparison group of the Control group-0 h vs. 200 mM NaCl-48 h, 56 genes were upregulated, and 36 genes were downregulated. Among them, the number of upregulated genes was POD (25), followed by GST (11), SOD (7), APX (6), GPX (4), CAT (2), and GR (1). The number of downregulated genes was POD (11), followed by SOD (9), GST (8), CAT (4), GR (2), APX (1), and GPX (1). In the 200 mM NaCl-48 h vs. 200 mM NaCl-168 h comparison group, 42 genes were upregulated, and 50 genes were downregulated. Among them, the most upregulated genes were SOD (12), followed by GST (8), POD (7), APX (5), CAT (4), GPX (4), and GR (2). The number of downregulated genes was POD (29), followed by GST (11), SOD (4), CAT (2), APX (2), GPX (1), and GR (1). In the Control group-0 h vs. 200 mM NaCl-168 h comparison group, 55 genes were upregulated, and 37 were downregulated. Among them, the number of upregulated genes was POD (16), followed by GST (12), SOD (10), APX (6), CAT (5), GPX (4), and GR (2). The number of downregulated genes was POD (20), followed by GST (7), SOD (6), CAT (1), APX (1), GPX (1), and GR (1). These results show that the roots of *T*. *ramosissima* were actively resisting NaCl stress by upregulating the expression of genes related to antioxidant enzyme activities within 168 h under NaCl stress.

According to the log_2_ fold-change analysis of *T*. *ramosissima* under NaCl stress for 48 and 168 h (Table 1), the expression levels of 14 DEGs were upregulated (Supplementary Figure S2). There were three genes in the SOD activity (*Unigene0096238*, *Unigene0027643*, and *Unigene0099972*), one gene in the POD activity (*Unigene0010026*), one gene in the CAT activity (*Unigene0089761*), four genes in the APX activity (*Unigene0105663*, *Unigene0105664, Unigene0008033,* and *Unigene0048032*), three genes in the GPX activity (*Unigene0019780*, *Unigene0026893*, and *Unigene0035407*), and two genes in the GST activity (*Unigene0015109* and *Unigene0020552*).

### 3.3. Analysis of KEGG Pathway in the Roots of T. ramosissima under NaCl Stress

We performed a KEGG pathway analysis of the top 10 in the comparison groups based on the Control group-0 h vs. 200 mM NaCl-48 h, 200 mM NaCl-48 h vs. 200 mM NaCl-168 h, and the Control group-0 h vs. 200 mM NaCl-168 h. We more intuitively observed the changes in the expression levels of DEGs and differential metabolites involved in the KEGG Pathway in *T*. *ramosissima*. In the Control group-0 h vs. 200 mM NaCl-48 h, 200 mM NaCl-48 h vs. 200 mM NaCl-168 h, and the Control group-0 h vs. 200 mM NaCl-168 h comparison groups (Table 2), we found that there were three identical KEGG pathways, namely, the flavonoid, phenylpropanoid, and zeatin biosynthesis pathways. Notably, the flavonoid biosynthesis pathway was significantly enriched with DEGs in the Control group-0 h vs. 200 mM NaCl-48 h, 200 mM NaCl-48 h vs. 200 mM NaCl-168 h, and the Control group-0 h vs. 200 mM NaCl -168 h comparison groups (*p* < 0.05).

### 3.4. Flavonoid Biosynthesis Pathway Analysis

**Figure 3:**
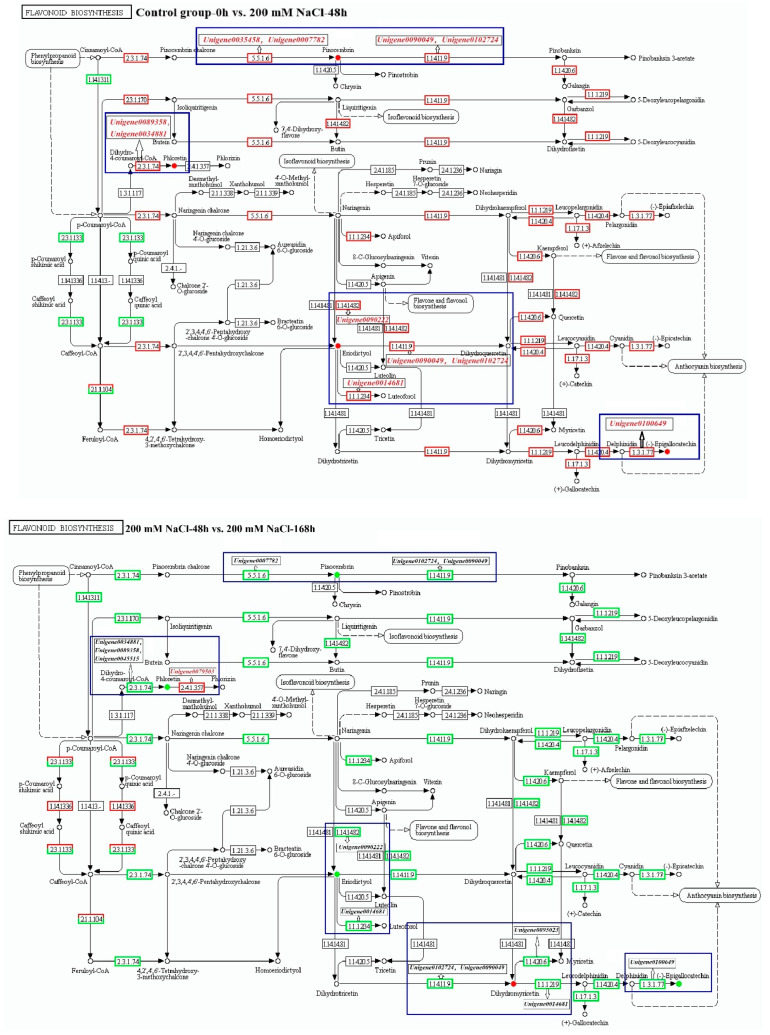

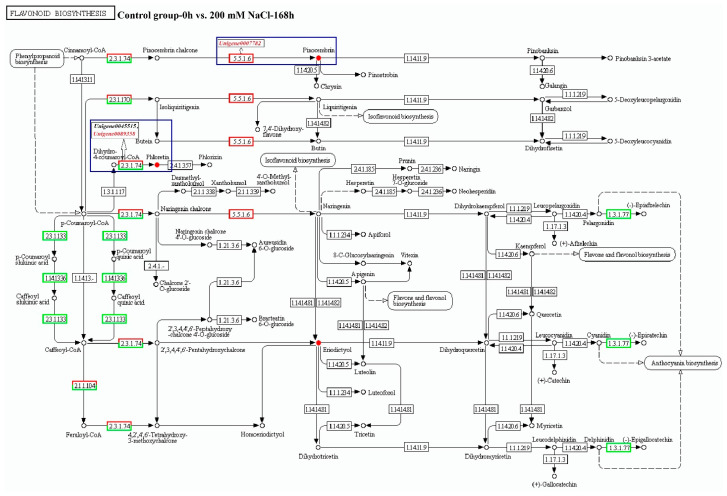
Flavonoid biosynthesis pathway annotated with DEGs and differential metabolites. (DEGs and metabolites annotated on the flavonoid biosynthesis pathway in the roots of *T*. *ramosissima* at 48 and 168 h under NaCl stress. Note: blue box: DEGs regulate their related differential metabolites; red genes: DEGs upregulated; black genes: DEGs downregulated; red differential metabolites: differential metabolite accumulation; green differential metabolites: differential metabolite degradation.

According to the previous results of Section 3.3, the flavonoid biosynthesis pathway was significantly enriched with DEGs in the A, B, and C comparison groups (*p* < 0.05), and these DEGs also regulated their associated differential metabolites (Figure 3). At 48 and 168 h under NaCl stress, we found five differential metabolites in the flavonoid biosynthesis pathway (Supplementary Table S2). Among them, four differential metabolites were found in the Control group-0 h vs. 200 mM NaCl-48 h comparison group, namely, pinocembrin, phloretin, eriodictyol, and (−)-epigallocatechin. Five differential metabolites were found in the 200 mM NaCl-48 h vs. 200 mM NaCl-168 h comparison group: pinocembrin, phloretin, eriodictyol, dihydromyricetin, and (−)-epigallocatechin. Three differential metabolites were found in the Control group-0 h vs. 200 mM NaCl-168 h comparison group, namely, pinocembrin, phloretin, and eriodictyol.

According to the analysis of the flavonoid biosynthesis pathway (Figure 3), we observed that *Unigene0035458* and *Unigene0007782* in the Control group-0 h vs. 200 mM NaCl-48 h comparison group positively regulated the pinocembrin accumulation upstream, and *Unigene0090049* and *Unigene0102724* were downstream and positively regulated the pinocembrin accumulation. *Unigene0089358* and *Unigene0034881* were upstream to positively regulate the accumulation of phloretin. *Unigene0090222* was upstream to positively regulate the accumulation of eriodictyol, and *Unigene0090049*, *Unigene0102724*, and *Unigene0014681* were downstream to positively regulate the accumulation of eriodictyol. *Unigene0100649* was in the upstream positive regulation of (−)-epigallocatechin accumulation. In the 200 mM NaCl-48 h vs. 200 mM NaCl-168 h comparison group, *Unigene0007782* was in the upstream and positively regulated the degradation of pinocembrin, whereas *Unigene0090049* and *Unigene0102724* were in the downstream and positively regulated the degradation of pinocembrin. *Unigene0090049* and *Unigene0102724* were downstream and positively regulated the degradation of pinocembrin. *Unigene0034881*, *Unigene0089358*, and *Unigene0045515* were located upstream to positively regulate the phloretin degradation, and *Unigene0079503* was in the downstream to negatively regulate the degradation of phloretin. *Unigene0090222* was in the upstream to positively regulate the degradation of eriodictyol, and *Unigene0090049*, *Unigene0102724*, and *Unigene0014681* were in the downstream to positively regulate the degradation of eriodictyol. *Unigene0090049* and *Unigene0102724* were in the upstream and negatively regulated the accumulation of dihydromyricetin, and *Unigene0095023* and *Unigene0014681* were in the downstream and negatively regulated the accumulation of dihydromyricetin. *Unigene0100649* was in the upstream positive regulation of (−)-epigallocatechin degradation. In the Control group-0 h vs. 200 mM NaCl-168 h comparison group, *Unigene0007782* was in the upstream and positively regulated the accumulation of pinocembrin. *Unigene0045515* was in the upstream and negatively regulated the accumulation of phloretin, whereas *Unigene0089358* was in the upstream and positively regulated the accumulation of phloretin. It is worth noting that in these three comparison groups, *Unigene0007782* was involved in the regulation of pinocembrin, and *Unigene0089358* was involved in regulating phloretin. *Unigene0007782* and *Unigene0089358* played important roles in the flavonoid biosynthesis pathway as candidate key genes for resistance to NaCl in *T*. *ramosissima*.

**Figure 4:**
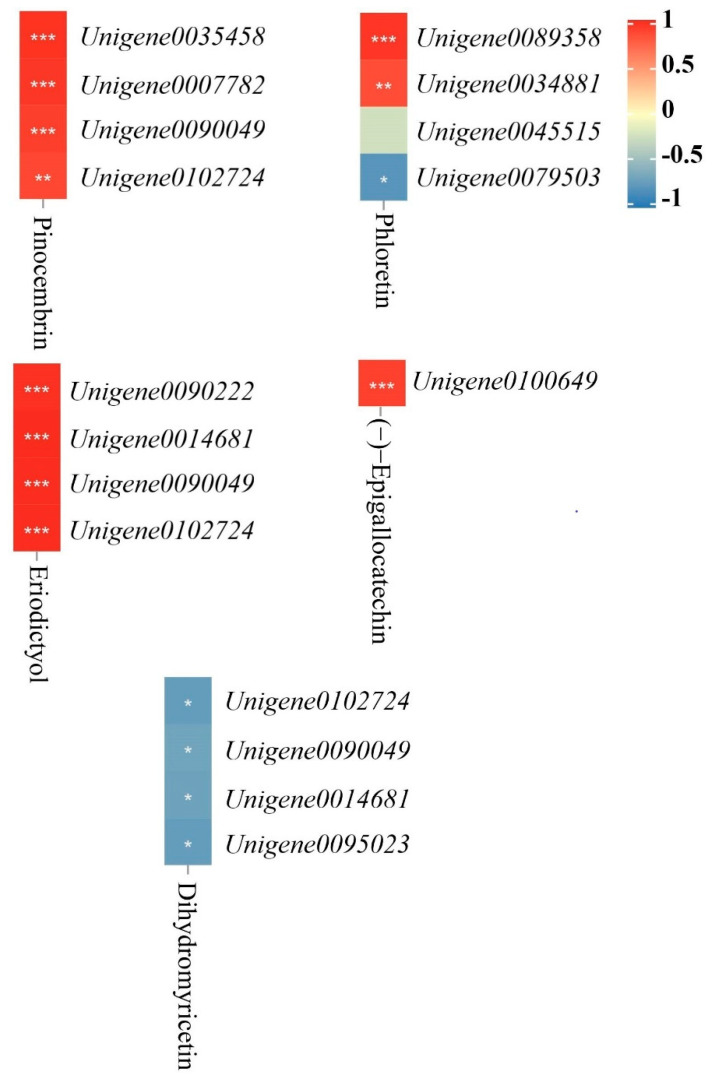
Heatmap of the correlation between DEGs and differential metabolites in the flavonoid biosynthesis pathway. (Heatmap of correlations between DEGs regulating differential metabolites in the flavonoid biosynthesis pathway and their differential metabolites. Note: *p* ≥ 0.05 is not marked; 0.01 < *p* < 0.05 is marked as *; 0.001 < *p* < 0.01 is marked as **; *p* ≤ 0.001 is marked as ***).

Additionally, according to the heatmap analysis of the correlation between the differential metabolites in the flavonoid biosynthesis pathway and their DEGs (Figure 4), we found that *Unigene0035458*, *Unigene0007782*, *Unigene0090049*, and *Unigene0102724* were significantly positively correlated with pinocembrin. *Unigene0089358* and *Unigene0034881* were significantly positively correlated with phloretin, and *Unigene0079503* was significantly negatively correlated with phloretin. *Unigene0090222*, *Unigene0014681*, *Unigene0090049*, and *Unigene0102724* were significantly positively correlated with eriodictyol. *Unigene0100649* was significantly positively correlated with (−)-epigallocatechin. *Unigene0102724*, *Unigene0090049*, *Unigene0014681*, and *Unigene0095023* were significantly negatively correlated with dihydromyricetin.

### 3.5. Phylogenetic Tree Analysis of Candidate Key Genes in the Flavonoid Biosynthesis Pathway

According to the results in 3.4, *Unigene0007782* and *Unigene0089358* were involved in regulating related metabolites in the flavonoid biosynthesis pathway to resist NaCl stress (Supplementary Table S3). Although the expression levels of *Unigene0007782* and *Unigene0089358* increased first and then decreased under NaCl stress at 48 and 168 h, the expression levels of *Unigene0007782* and *Unigene0089358* at 168 h were significantly higher than those of the control group (Supplementary Figure S3). Therefore, *Unigene0007782* and *Unigene0089358* were selected as candidate key genes in the flavonoid biosynthesis pathway. Using Blast, we selected the protein amino acid sequences of *Unigene0007782* and *Unigene0089358* for alignment on NCBI. Then, 15 homologous gene species of *Unigene0007782* (Supplementary Table S4) and 15 homologous gene species of *Unigene0089358* (Supplementary Table S5) were selected. Finally, we used MEGA software (MEGA Software, Coatesville, USA) to construct a phylogenetic tree by combining the protein amino acid sequences of *Unigene0007782* and *Unigene0089358* of *T*. *ramosissima* with their respective 15 homologous gene species protein amino acid sequences. The results show that *Unigene0007782* was closely related to *Beta vulgaris* subsp. *vulgaris* (Supplementary Figure S4), and *Unigene0089358* was also closely related to *Beta vulgaris* subsp. *vulgaris* (Supplementary Figure S5).

### 3.6. Quantitative Real-Time PCR (qRT-PCR) Validation of Candidate Key Genes

**Figure 5:**
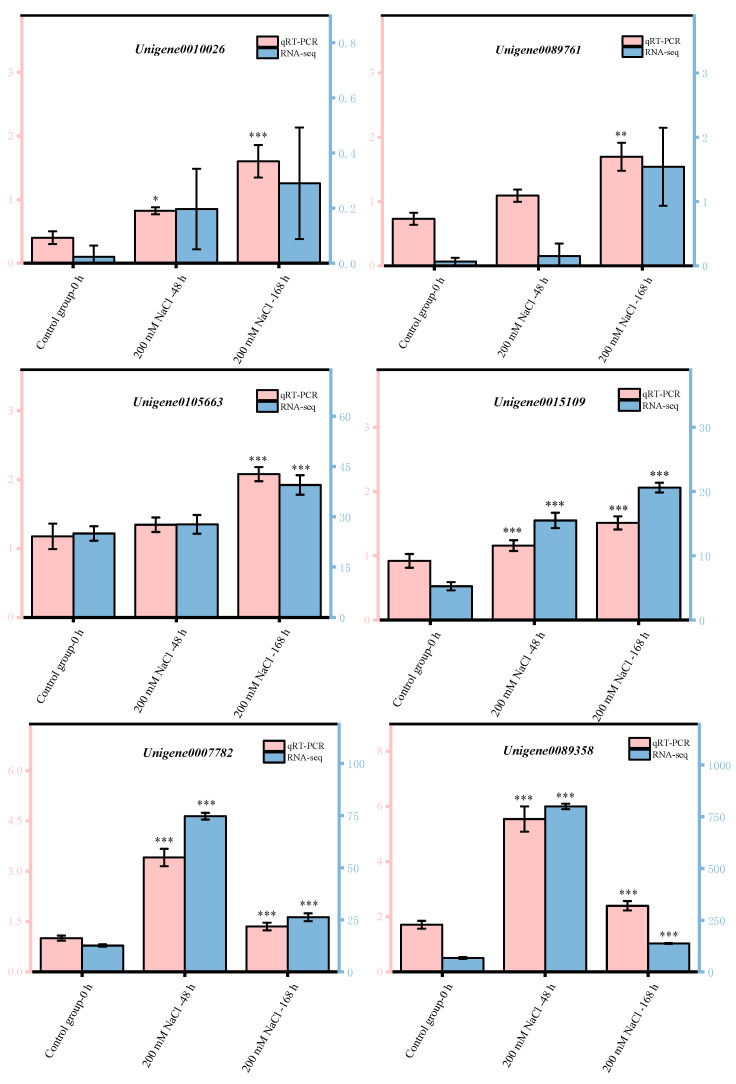

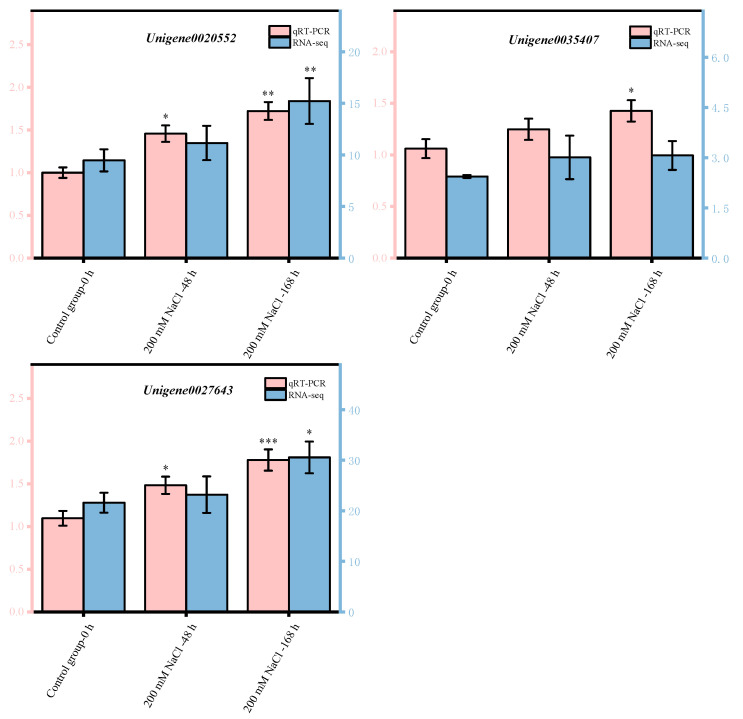
qRT-PCR validation of candidate key genes. (Nine candidate key genes were obtained based on antioxidant enzyme activity and gene expression in the flavonoid biosynthesis pathway. Note: *p* ≥ 0.05 is not marked; 0.01 < *p* < 0.05 is marked as *; 0.001 < *p* < 0.01 is marked as **; *p* ≤ 0.001 is marked as ***; red color: numerical value is shown on the left side of the *Y* axis; blue color: numerical value is shown on the right side of the *Y* axis).

We selected nine candidate key genes obtained in this study for qRT-PCR validation. Notably, the qRT-PCR validation results in this study were in complete agreement with the expression trends of transcriptome sequencing analysis (Figure 5). Therefore, the transcriptome data obtained in this study are accurate. These findings can provide a scientific theoretical basis for mining candidate key genes for improving salt tolerance and alleviating NaCl stress injury in *T*. *ramosissima*.

## 4. Discussion

The effects of salt stress on plant growth and development [52] are well-documented. Excess salinity in soil can be characterized by the excessive accumulation of sodium chloride, which inhibits plants’ absorption of water and nutrients from the soil, ultimately leading to slow plant growth and eventual plant death [53,54]. When plants are subjected to salt stress, the integrity of the plants’ cell membranes will be destroyed, the ions will be unbalanced, and ROS will eventually be generated [55]. Excessive accumulation of ROS can lead to oxidative stress, which can damage biological macromolecules, biofilms, and other structures and even lead to cell death [43]. It is noteworthy that salt-induced oxidative stress has the greatest effect on the root tissue of plants [56,57].

H_2_O_2_ is the most common ROS molecule, and H_2_O_2_ can also act as a signal transduction molecule to activate plant defense responses [58]. MDA is a product of lipid peroxidation and accumulates when oxidative stress is particularly severe [59]. It is worth noting that lipid peroxidation is an indicator of oxidative damage caused by salt stress, and the MDA content marks the degree of damage to plant cell membranes [60,61]. In this study, the content of H_2_O_2_ and MDA in the roots of *T*. *ramosissima* increased under NaCl stress for 48 and 168 h. The results show that the roots of *T*. *ramosissima* were affected by NaCl, ROS accumulated, and the oxidative stress was intense, which might cause cell membrane damage.

Furthermore, halophytes have evolved an efficient ROS scavenging system [62,63]. In the antioxidant defense mechanism, SOD is able to catalyze O_2_^−^ to H_2_O_2_, and CAT further converts this H_2_O_2_ into H_2_O and O_2_. POD is a key enzyme that scavenges H_2_O_2_ during oxidative stress in plants [57,64,65]. In addition, under salt stress, plants can use antioxidant enzymes such as SOD, POD, and CAT to scavenge the excessive accumulation of ROS, thereby reducing the damage to plants [66]. The upregulation of SOD, POD, and CAT activities and other related gene expression levels will promote the enhancement of enzyme activity, the ability to scavenge ROS, and ultimately improve the resistance of plants to abiotic stress [67]. In this study, the SOD, POD, and CAT activities in *T*. *ramosissima* roots increased with time under NaCl stress for 168 h, and in the Control group-0 h vs. 200 mM NaCl-168 h comparison group, the SOD activity and CAT activity-related genes were upregulated and mainly involved in related metabolic pathways to resist NaCl stress. However, the POD activity-related genes showed more downregulated genes than upregulated genes in the Control group-0 h vs. 200 mM NaCl-168 h comparison group, and more upregulated genes than downregulated genes in the Control group-0 h vs. 200 mM NaCl-48 h comparison group. The results show that the POD activity was actively involved in the relevant metabolic pathways to resist NaCl stress during 48 h of NaCl stress. However, the related genes in the POD activity were partially downregulated due to the prolonged NaCl stress. APX, GPX, and GR are important enzymes for scavenging intracellular ROS [68].

Meanwhile, APX is able to maintain the optimal level of H_2_O_2_ as a signaling molecule [69]. APX is considered to be one of the main factors in reducing free radicals such as H_2_O_2_, which can minimize plant damage caused by oxidative stress [70]. APX and GPX can also convert H_2_O_2_ to H_2_O and O_2_ [71]. Moreover, increased GR activity can maintain the ratio of GSH/GSH + GSSG at an optimal level, thereby improving plant tolerance to abiotic stress [57]. Compared to barley treated with 200 mM of salt, salt-tolerant barley showed less oxidative damage and higher antioxidant enzyme activities, such as SOD, APX, GPX, and GR [72]. In this study, the expression levels of APX activity, GPX activity, and GR activity-related genes were mainly upregulated in the roots of *T*. *ramosissima* under NaCl stress for 168 h. This indicates that APX, GPX, and GR were all involved in the plant response to NaCl stress, and their upregulation of related genes alleviated the damage caused by NaCl stress. The role of GST is detoxification and antioxidant defense. It can protect plants from different adversities [73]. Studies have shown that GST-active gene expression levels are sensitive to concentrations above 200 mM of NaCl but insensitive to concentrations below 100 mM of NaCl [74]. Under NaCl stress, the expression levels of GST activity-related genes in tobacco were upregulated, which improved the salt tolerance of tobacco [75]. Under NaCl stress, gene expression levels of antioxidant enzymes were mainly upregulated within 168 h in *T. ramosissima*. In particular, 14 genes in antioxidant enzyme activities were upregulated at 48 and 168 h under NaCl stress, which helped *T*. *ramosissima* to resist NaCl stress, and they participated in the related KEGG pathway under NaCl stress for 48 and 168 h and actively resisted NaCl stress. They play an important role in improving the salt tolerance of *T*. *ramosissima*. In this study, *T*. *ramosissima* resists salt poisoning by enhancing the antioxidant defense system, which is similar to the previous study [76,77,78].

Flavonoid biosynthesis and antioxidant pathways play important roles in plant defense during NaCl stress [79]. Flavonoids can eliminate ROS accumulation to improve plants’ tolerance to stress and alleviate the damage caused by unfavorable factors [80,81,82]. In addition, flavonoids are a kind of polyphenolic compound [83]. In many halophytes, increased salt tolerance is positively associated with increased phenolic compounds [84]. Phenolic compounds produced by plant metabolism also play a role in improving plant salt tolerance [85]. Phloretin, a dihydrochalcone flavonoid, exhibits potent antioxidant activity in scavenging and inhibiting lipid peroxidation [86]. Phloretin formation depends on the chalcone synthase family [87]. Chalcone synthase (CHS) is the key and first rate-limiting enzyme in the flavonoid biosynthesis pathway [88]. Studies have shown that the overexpression of *EaCHS1* in *Eupatorium adenophorum* increases the downstream flavonoid production and expression of related genes in the phenylpropanoid pathway, and the elevated expression level of *EaCHS1* increases the accumulation of flavonoids in plants and improves the tolerance of plants to salt stress [89]. The overexpression of *AeCHS* in *Abelmoschus esculentus* increases the downstream flavonoid production and expression of related genes in flavonoid biosynthesis pathways and enhances salt resistance during root development [90]. *Unigene0089358* is a gene related to chalcone synthase. Its expression was upregulated in the comparison group of the Control group-0 h vs. 200 mM NaCl-48 h and the Control group-0 h vs. 200 mM NaCl-168 h, which has been in the upstream of phloretin and positively regulates the accumulation of phloretin. There is a significant positive correlation between *Unigene0089358* and phloretin. Pinocembrin is a major flavonoid molecule and one of the major flavonoids [91]; it shows an antioxidant effect [92]. Studies have shown pinocembrin as a salinity stress reliever to reduce NaCl damage to okra plants [93]. Chalcone isomerase (CHI) is the second key rate-limiting enzyme in the flavonoid biosynthesis pathway [94]. It exists only in plants [95]. CHI-related genes in *Millettia pinnata* help yeast transformants reject the entry of salt ions, thereby enhancing salt tolerance [96]. CHI-related genes involved in flavonoid metabolite production were upregulated under NaCl treatment in the roots of *Medicago sativa* [97].

## 5. Conclusions

In conclusion, through the analysis of the physiology, transcriptome, and metabolites of *T*. *ramosissima* root under NaCl stress, our results suggest that enzymatic and nonenzymatic antioxidant defense systems can help scavenge ROS and maintain and enhance plant growth and development when plants are under NaCl stress. This study elucidates the changes in antioxidant enzyme activities in the roots of *T*. *ramosissima* under NaCl stress and unearths candidate key genes and metabolic pathways related to the antioxidant mechanism. It provides a scientific theoretical basis for studying the molecular mechanism and salt tolerance gene resources of *Tamarix* plants to alleviate salt poisoning and lays a foundation for breeding forest tree varieties on saline–alkali land.

## Figures and Tables

**Table 1:** Antioxidant enzyme activity differentially expressed genes annotated to KEGG pathway.

Pathway	Gene ID	Description	Log_2_ Fold-Change		
			Control Group-0 h vs. 200 mM NaCl-48 h	200 mM NaCl-48 h vs. 200 mM NaCl-168 h	Control Group-0 h vs. 200 mM NaCl168 h
SOD					
ko04146	*Unigene0022482*	SOD	3.71	−2.85	0.86
	*Unigene0033269*	SOD4 protein, partial	−0.07	1.14	1.07
	*Unigene0096238*	SOD	5.64	5.28	10.92
	*Unigene0023980*	Iron/manganese superoxide dismutase	−2.37	1.12	−1.25
	*Unigene0027643*	Mn superoxide dismutase	0.10	0.40	0.50
	*Unigene0032758*	Superoxide dismutase, Fe-Mn family	−0.42	2.11	1.69
	*Unigene0033135*	Manganese superoxide dismutase	−1.91	5.47	3.55
	*Unigene0034506*	Superoxide dismutase	−3.98	1.66	−2.31
	*Unigene0035818*	Superoxide dismutase	−10.88	6.74	−4.15
	*Unigene0050462*	Superoxide dismutase	−0.72	1.58	0.86
	*Unigene0059078*	Cu Zn superoxide dismutase	−6.38	8.26	1.88
	*Unigene0074194*	Mitochondrial Mn superoxide dismutase	2.10	−8.00	−5.91
	*Unigene0082550*	Superoxide dismutase	−0.91	1.02	0.11
	*Unigene0099972*	Superoxide dismutase [Cu-Zn]	5.54	4.02	9.56
	*Unigene0104234*	Superoxide dismutase [Cu-Zn] 1	0.08	−0.20	−0.11
	*Unigene0002762*	Copper/zinc superoxide dismutase	11.34	−11.34	0.00
POD					
ko01100; ko01110; ko00940	*Unigene0000894*	Peroxidase 27-like	2.37	−0.85	1.52
	*Unigene0002490*	Peroxidase 64-like	0.46	−1.72	−1.26
	*Unigene0004177*	Peroxidase 3-like	0.85	−0.93	−0.08
	*Unigene0009260*	Peroxidase 20	0.14	−1.46	−1.32
	*Unigene0010026*	Peroxidase 29-like	3.08	0.56	3.64
	*Unigene0013825*	Peroxidase	2.06	−2.03	0.03
	*Unigene0013827*	Peroxidase	1.25	−0.82	0.42
	*Unigene0014843*	Peroxidase	−2.26	0.92	−1.34
	*Unigene0016209*	Peroxidase 27-like	1.49	−1.82	−0.34
	*Unigene0021987*	Peroxidase 27-like	0.00	−1.82	−1.82
	*Unigene0022473*	Peroxidase A2-like	0.16	−0.47	−0.31
	*Unigene0029752*	Peroxidase 17	0.42	−0.72	−0.31
	*Unigene0033992*	Peroxidase 3-like	−0.51	−1.16	−1.68
	*Unigene0033993*	Peroxidase 3-like	2.10	−2.02	0.07
	*Unigene0034947*	Peroxidase 73-like	5.05	−3.55	1.50
	*Unigene0049353*	Peroxidase 5	−3.20	4.08	0.87
	*Unigene0052502*	Peroxidase 72	−1.20	1.20	0.00
	*Unigene0053312*	Peroxidase 72-like	−1.30	0.42	−0.89
	*Unigene0053707*	Peroxidase 27	2.88	−1.85	1.03
	*Unigene0058419*	Peroxidase P7-like	−0.91	0.63	−0.28
	*Unigene0064700*	Peroxidase 4	0.99	−0.21	0.78
	*Unigene0068179*	Peroxidase 60-like	6.75	−4.34	2.41
	*Unigene0076135*	Peroxidase P7	−0.34	1.13	0.80
	*Unigene0076259*	Peroxidase 5-like	−0.08	−0.50	−0.58
	*Unigene0076940*	Peroxidase 60-like	−2.20	−1.01	−3.21
	*Unigene0079101*	Peroxidase P7	1.48	−0.27	1.21
	*Unigene0080006*	Peroxidase 57-like	1.96	−3.18	−1.23
	*Unigene0084405*	Peroxidase N-like	0.55	−0.63	−0.08
	*Unigene0084406*	Peroxidase N	0.96	−0.57	0.39
	*Unigene0087484*	Peroxidase 11-like	0.13	−0.89	−0.76
	*Unigene0089435*	Peroxidase 73-like	0.06	−0.31	−0.25
	*Unigene0090964*	Peroxidase	−0.13	−1.00	−1.13
	*Unigene0092878*	Peroxidase 3-like	1.37	−1.62	−0.25
	*Unigene0094375*	Peroxidase 31	1.93	−0.53	1.39
	*Unigene0102899*	Peroxidase 7	5.12	−3.58	1.54
	*Unigene0104832*	Peroxidase P7-like	2.75	−3.45	−0.70
CAT					
ko01100; ko01110; ko01200; ko00630; ko04146; ko04016; ko00380	*Unigene0038031*	Peroxisomal catalase-like	−7.52	0.00	−7.52
	*Unigene0046159*	Catalase isozyme 1	−0.11	0.19	0.08
	*Unigene0046160*	Catalase, partial	−0.93	1.40	0.47
	*Unigene0052968*	Catalase	6.87	−2.32	4.54
	*Unigene0068901*	Catalase	−1.09	3.23	2.13
	*Unigene0089761*	Catalase 3	1.20	3.33	4.53
APX					
ko01100; ko00480; ko00053	*Unigene0105663*	Thylakoid-bound ascorbate peroxidase, partial	0.15	0.51	0.66
	*Unigene0105664*	Thylakoid ascorbate peroxidase precursor, partial	1.09	0.37	1.46
	*Unigene0008032*	L-ascorbate peroxidase 3	−0.27	0.43	0.16
	*Unigene0008033*	L-ascorbate peroxidase 3	0.23	0.30	0.54
	*Unigene0008513*	Peroxidase domain-containing protein	2.67	−3.21	−0.53
	*Unigene0048032*	L-ascorbate peroxidase, cytosolic-like	0.29	0.51	0.80
	*Unigene0048033*	Cytosolic ascorbate peroxidase	1.24	−0.57	0.67
GPX					
ko01100; ko00480; ko00590	*Unigene0001490*	Glutathione peroxidase 2 family protein	0.50	−0.61	−0.11
	*Unigene0019780*	Glutathione peroxidase 1	7.62	3.38	11.00
	*Unigene0026893*	Glutathione peroxidase	6.13	6.37	12.50
	*Unigene0035407*	Glutathione peroxidase	0.30	0.03	0.33
	*Unigene0103632*	Glutathione peroxidase	−6.20	8.71	2.52
GST					
ko01100; ko00480	*Unigene0071136*	Glutathione S-transferase-like	−0.82	−0.74	−1.56
	*Unigene0071137*	Glutathione S-transferase-like	−0.01	0.23	0.22
	*Unigene0076278*	Glutathione S-transferase U8-like	0.10	−1.47	−1.37
	*Unigene0076819*	Glutathione S-transferase F9	−6.38	8.31	1.93
	*Unigene0081745*	Glutathione S-transferase U10-like	0.28	−0.47	−0.19
	*Unigene0082147*	Glutathione S-transferase F11-like	3.43	−3.61	−0.18
	*Unigene0098941*	Glutathione S-transferase U9	−3.13	1.18	−1.95
	*Unigene0001041*	Glutathione S-transferase	0.12	−10.74	−10.62
	*Unigene0004890*	Glutathione S-transferase T1-like	−0.40	−0.64	−1.04
	*Unigene0007072*	Glutathione S-transferase U17-like	1.14	−0.73	0.42
	*Unigene0015109*	Glutathione S-transferase U8-like	1.56	0.41	1.97
	*Unigene0020552*	Glutathione S-transferase	0.24	0.45	0.68
	*Unigene0040702*	Glutathione S-transferase 4-like	−6.06	4.91	−1.15
	*Unigene0048538*	Glutathione S-transferase U10-like	−1.76	0.77	−0.99
	*Unigene0049699*	Glutathione S-transferase F3-like	−9.86	8.04	−1.82
	*Unigene0056773*	Glutathione S-transferase	1.00	−1.73	−0.74
	*Unigene0064942*	Glutathione S-transferase L3	0.89	−0.56	0.33
	*Unigene0069058*	Glutathione-S-transferase	1.04	−0.21	0.83
	*Unigene0069060*	Glutathione S-transferase L3-like	1.11	−1.21	−0.10
GR					
ko01100; ko00480	*Unigene0056650*	Glutathione reductase	−8.31	7.82	−0.48
	*Unigene0056651*	Glutathione reductase	−3.15	4.14	0.99
	*Unigene0075696*	Glutathione reductase	0.86	−0.72	0.14

**Table 2:** Top 10 KEGG pathways.

ID	Name	Genes Numbers	*p*-Value	Metabolites Numbers	*p*-Value	KEGG Pathway
Control group-0 h- vs. 200 mM NaCl-48 h						
1	Flavonoid biosynthesis	24	0.000000	4	0.574614	ko00941
2	Phenylpropanoid biosynthesis	65	0.000301	6	0.031299	ko00940
3	Zeatin biosynthesis	9	0.025143	2	0.243249	ko00908
4	Carbon fixation in photosynthetic organisms	108	0.040218	1	0.519880	ko00710
5	Biotin metabolism	12	0.145600	2	0.049622	ko00780
6	Steroid biosynthesis	26	0.217384	1	0.443531	ko00100
7	Isoflavonoid biosynthesis	1	0.218511	2	0.453899	ko00943
8	Cysteine and methionine metabolism	95	0.319950	2	0.629016	ko00270
9	Linoleic acid metabolism	7	0.335061	1	0.519880	ko00591
10	Ubiquinone and other terpenoid–quinone biosynthesis	21	0.384894	2	0.403533	ko00130
200 mM NaCl-48 h- vs. 200 mM NaCl-168 h						
1	Flavonoid biosynthesis	24	0.000001	5	0.229149	ko00941
2	Zeatin biosynthesis	13	0.000166	2	0.186623	ko00908
3	Phenylpropanoid biosynthesis	69	0.000178	2	0.665973	ko00940
4	Cysteine and methionine metabolism	106	0.140185	3	0.241698	ko00270
5	Linoleic acid metabolism	8	0.241007	1	0.458597	ko00591
6	Starch and sucrose metabolism	98	0.305304	2	0.276678	ko00500
7	Ubiquinone and other terpenoid–quinone biosynthesis	23	0.315237	1	0.709419	ko00130
8	Carbon fixation in photosynthetic organisms	102	0.356294	1	0.458597	ko00710
9	Valine, leucine, and isoleucine biosynthesis	20	0.418018	1	0.387488	ko00290
10	Monobactam biosynthesis	10	0.419637	1	0.577450	ko00261
Control group-0 h- vs. 200 mM NaCl-168 h						
1	Phenylpropanoid biosynthesis	53	0.000000	6	0.051241	ko00940
2	Flavonoid biosynthesis	11	0.010488	3	0.847245	ko00941
3	Zeatin biosynthesis	6	0.044825	2	0.287087	ko00908
4	Tryptophan metabolism	30	0.055480	2	0.462729	ko00380
5	Pentose and glucuronate interconversions	23	0.190093	1	0.562093	ko00040
6	Glutathione metabolism	43	0.210565	1	0.686318	ko00480
7	Monobactam biosynthesis	7	0.232453	1	0.686318	ko00261
8	Cutin, suberine, and wax biosynthesis	5	0.344352	1	0.389840	ko00073
9	Fatty acid elongation	12	0.367798	1	0.151436	ko00062
10	Nitrogen metabolism	20	0.399368	1	0.151436	ko00910

## Data Availability

Not applicable.

## Supplementary Materials

The following supporting information can be downloaded at: https://www.mdpi.com/article/10.3390/antiox11122362/s1, Supplementary Table S1: The sequences of specific primers. Supplementary Table S2: Differential metabolites in flavonoid biosynthesis pathway. Supplementary Table S3: Candidate key genes in the flavonoid biosynthesis pathway. Supplementary Table S4: Fifteen homologous species Information Sheet for *Unigene0007782*. Supplementary Table S5: Fifteen homologous species Information Sheet for *Unigene0089358*. Supplementary Figure S1: Statistical chart of the expression number of key genes of antioxidant enzymes in the roots of *T. ramosissima* under NaCl stress. Supplementary Figure S2: Expression levels of differentially expressed genes related to antioxidant enzyme activity. Supplementary Figure S3: The expression levels of candidate key genes in flavonoid biosynthesis pathway. Supplementary Figure S4: Phylogenetic tree analysis of *T. ramosissima* chalcone-flavonone isomerase and other species chalcone-flavonone isomerase. Supplementary Figure S5: Phylogenetic tree analysis of *T. ramosissima* chalcone synthase and other species chalcone synthase.
