# Supplementary material for: Analysis of the Antioxidant Mechanism of Tamarix ramosissima Roots under NaCl Stress Based on Physiology, Transcriptomic and Metabolomic
Source: Antioxidants (Basel). 2022 Nov 28;11(12):2362. doi: 10.3390/antiox11122362 (PMC9774368; doi:10.3390/antiox11122362)
Supplement: Supplementary file 1 [file antioxidants-11-02362-s001.zip › antioxidants-2047677-supplementary.pdf]

Supplementary Table S1. The sequences of specific primers

ID	Primer Name	Primer Sequence (5'-3')
1	*Unigene0010026*	F: CAAGTCTTCGTGTCCGTATGTGGAA<br>R: CAGAAGCAGTAGCGTCGGTTGATAA
2	*Unigene0089761*	F: AAGGATCTCCACGATGCCATTGC<br>R: CCTGTTCAGAACCAAGCGACCAA
3	*Unigene0105663*	F: TGAGCCAGAGCAGTGTCCAGAA<br>R: CCAGCCACTTCGTTCAGGTCTTG
4	*Unigene0015109*	F: TCATGCCTCAAGACCCATACAACAG<br>R: CGTGCTTCCTCTTCACCGTTCC
5	*Unigene0007782*	F: GCTGCTGCCGAGATTGTGATTAGT<br>R: GACAACTTGGCGGAGAGGTTATGG
6	*Unigene0089358*	F: CGCAATGTGCTGAGTGAGTATGGT<br>R: CAACAGTAAGACCAGGACCGAACC
7	*Unigene0020552*	F: TGAGGACATAGTGCTTGCTGATTCG<br>R: TTGAAGTGGCTGTATGCTTGAGGAA
8	*Unigene0035407*	F: TGGTAGTGGTGTTACGAGGTCAAAC<br>R: TGTTCATCACTTCCTGGCTCTTGC
9	*Unigene0027643*	F: GGAAGAACCTTGCTCCTGTCAATCA<br>R: ACTCTTTATCCACACCAAGCCACAC
10	*Tubulin*	F: GCTGAGATTACAACCGCTG<br>R: CTGTTCGTTTGGTCTTGATT

Note: F means sense primer; R means anti-sense primer.

Supplementary Table S2. Differential metabolites in flavonoid biosynthesis pathway

Name	Formula	Molecular Weight	PPM	RT [min]	m/z	Control group-0h-1	Control group-0h-2	Control group-0h-3	NaCl-48h-1	NaCl-48h-2	NaCl-48h-3	NaCl-168h-1	NaCl-168h-2	NaCl-168h-3
Pinocembrin	$C_{15}H_{12}O_4$	256.07	2.61	10.59	257.08	27.22	27.08	27.48	30.76	30.73	31.00	29.79	29.92	29.85
Phloretin	$C_{15}H_{14}O_5$	274.08	1.40	7.68	275.09	24.66	24.83	24.75	27.21	26.99	26.90	26.04	25.71	26.11
Eriodictyol	$C_{15}H_{12}O_6$	288.06	1.31	10.05	289.07	24.21	24.14	24.79	30.00	30.06	29.88	26.56	26.98	26.76
(−)−Epigallocatechin	$C_{15}H_{14}O_7$	288.06	58845.59	6.10	289.07	22.73	22.11	23.92	25.98	25.73	25.36	24.31	24.26	24.12
Dihydromyricetin	$C_{15}H_{12}O_8$	320.06	11.66	6.56	365.06	24.18	24.24	23.72	22.46	22.77	22.60	25.13	25.52	25.01

Note: All of the data inside the table are relative quantitative values, no unit required.

Supplementary Table S3. Candidate key genes in the flavonoid biosynthesis pathway

		Log$_2$ fold-change		
Gene ID	Description	Control group-0h vs. 200 mM NaCl-48h	200 mM NaCl-48h vs. 200 mM NaCl -168h	Control group-0h vs. 200 mM NaCl-168h
Unigene0007782	Chalcone synthase	2.56	-1.51	1.05
Unigene0089358	Predicted: probable chalcone-flavonone isomerase 3	3.58	-2.54	1.04

Supplementary Table S4. 15 homologous species Information Sheet for *Unigene0007782*

Family	Species	Description	Protein ID	CDS (bp)	ORF length (aa)
Asteraceae	*Lactuca sativa*	Chalcone isomerase-like protein 2	XP_023770992.1	621	206
Proteaceae	*Macadamia integrifolia*	Chalcone isomerase-like protein 2	XP_042503463.1	633	210
Ericaceae	*Vaccinium corymbosum*	Chalcone isomerase	AYC35388.1	630	209
Euphorbiaceae	*Hevea brasiliensis*	Probable chalcone-flavonone isomerase 3	XP_021684778.1	630	209
Proteaceae	*Telopea speciosissima*	Chalcone isomerase-like protein 2 isoform X1	XP_043723638.1	630	209
Amaranthaceae	*Beta vulgaris* subsp. *vulgaris*	Chalcone isomerase-like protein 2 isoform X1	XP_048494133.1	633	210
Asteraceae	*Tanacetum cinerariifolium*	Chalcone isomerase	GEW79335.1	621	206
Caricaceae	*Carica papaya*	Probable chalcone-flavonone isomerase 3 isoform X1	XP_021904134.1	636	211
Asteraceae	*Erigeron canadensis*	Chalcone isomerase-like protein 2	XP_043636289.1	633	210
Fagaceae	*Quercus suber*	Probable chalcone-flavonone isomerase 3	XP_023907747.1	630	209
Fagaceae	*Quercus lobata*	Probable chalcone-flavonone isomerase 3	XP_030932490.1	630	209
Euphorbiaceae	*Manihot esculenta*	Chalcone isomerase-like protein 2	XP_021617615.1	630	209
Theaceae	*Camellia sinensis*	Probable chalcone-flavonone isomerase 3	XP_028070241.1	630	209
Theaceae	*Camellia fraterna*	Chalcone isomerase	AUT30534.1	630	209
Rosaceae	*Prunus persica*	Probable chalcone-flavonone isomerase 3	XP_007218407.1	630	209

Supplementary Table S5. 15 homologous species Information Sheet for *Unigene0089358*

Family	Species	Description	Protein ID	CDS (bp)	ORF length (aa)
Amaranthaceae	*Beta vulgaris* subsp. *vulgaris*	Chalcone synthase	XP_010682889.2	1179	392
Polygonaceae	*Fagopyrum tataricum*	Chalcone synthase	ACZ51475.1	1188	395
Nelumbonaceae	*Nelumbo nucifera*	Predicted: chalcone synthase	XP_010249317.1	1182	393
Asteraceae	*Gynura bicolor*	Chalcone synthase	BAJ17656.1	1197	398
Sapindaceae	*Koelreuteria paniculata*	Chalcone synthase	QXN66168.1	1182	393
Asteraceae	*Chamaemelum nobile*	Chalcone synthase	ASW41112.1	1200	399
Lythraceae	*Punica granatum*	Chalcone synthase	XP_031381600.1	1197	398
Sapindaceae	*Dimocarpus longan*	Chalcone synthase	AEO36981.1	1182	393
Malvaceae	*Hibiscus syriacus*	Chalcone synthase	XP_039069650.1	1191	396
Vitaceae	*Vitis riparia*	Chalcone synthase-like	XP_034707297.1	1182	393
Vitaceae	*Nekemias grossedentata*	Chalcone synthase	QUE49096.1	1182	393
Anacardiaceae	*Pistacia vera*	chalcone synthase 2	XP_031255866.1	1194	397
Meliaceae	*Dysoxylum binectariferum*	Chalcone synthase	AMR58953.1	1188	395
Rutaceae	*Citrus sinensis*	Chalcone synthase 2	NP_001306986.1	1176	391
Myrtaceae	*Syzygium oleosum*	Chalcone synthase	XP_030450429.1	1191	396

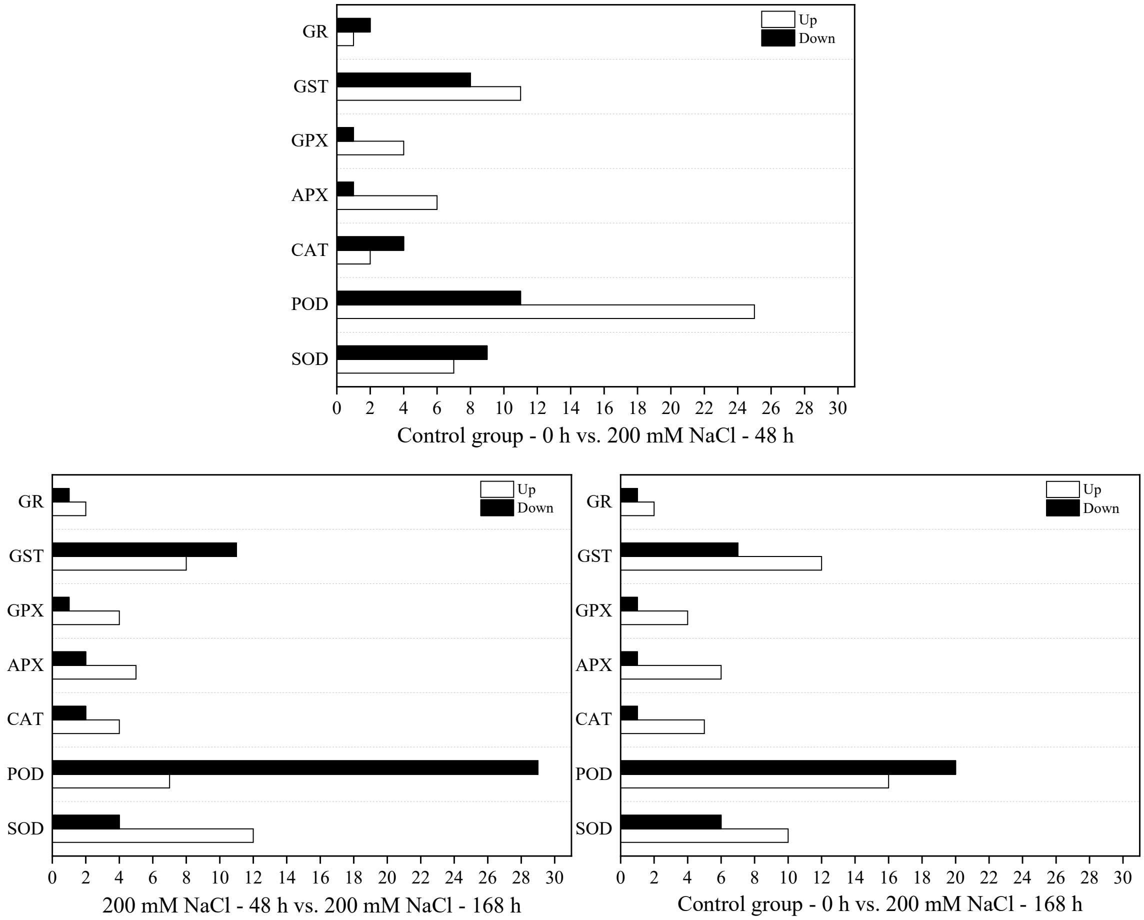

Supplementary Figure S1. Statistical chart of the expression number of key genes of antioxidant enzymes in the roots of *T. ramosissima* under NaCl stress

(The number of up- and down-regulated expression of candidate genes for antioxidant enzyme activities in roots of *T. ramosissima* under NaCl stress. Note: SOD: superoxide dismutase; POD: peroxidase; CAT: catalase; APX: ascorbic acid peroxidase; GPX: glutathione peroxidase; GST: glutathione S-transferase; GR: glutathione reductase).

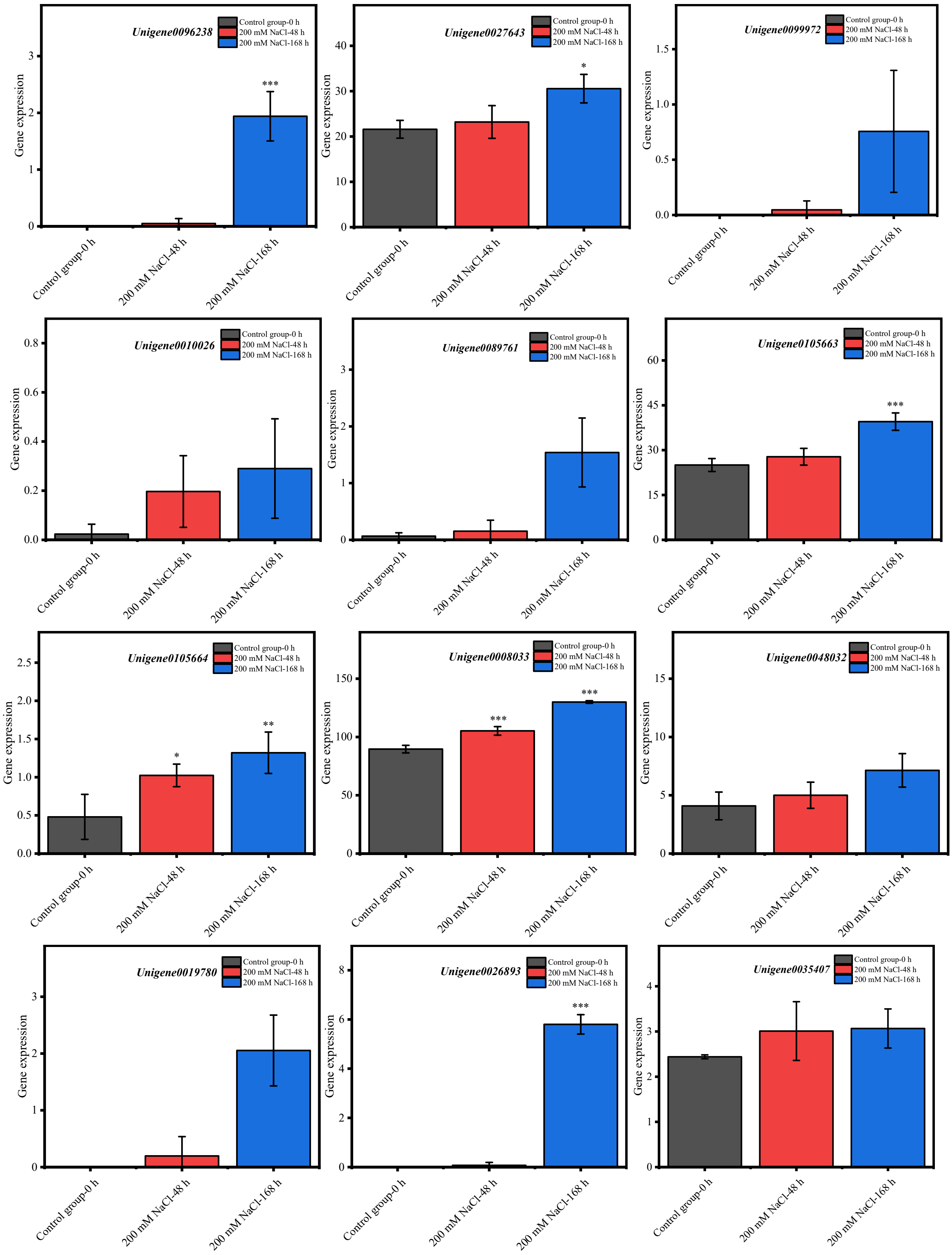

Unigene0096238
Unigene0027643
Unigene0099972
Unigene0010026
Unigene0089761
Unigene0105663
Unigene0105664
Unigene0008033
Unigene0048032
Unigene0019780
Unigene0026893
Unigene0035407
Control group-0 h
200 mM NaCl-48 h
200 mM NaCl-168 h
Gene expression

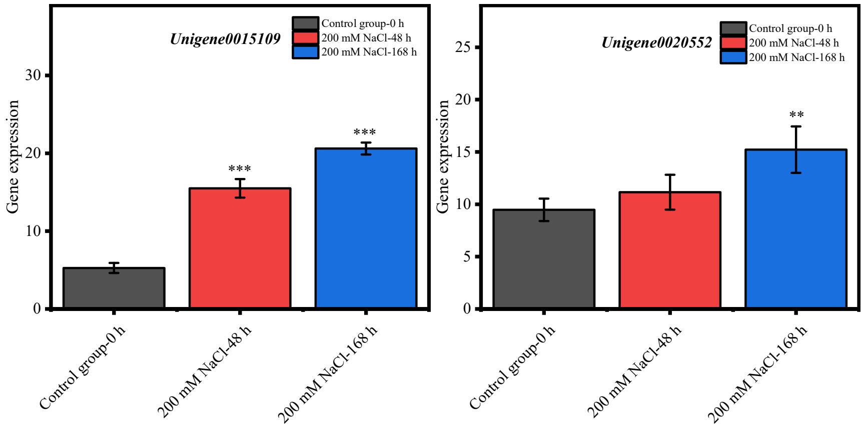

Supplementary Figure S2. Expression levels of differentially expressed genes related to antioxidant enzyme activity (The expression levels of 14 differentially expressed genes related to antioxidant enzyme activity in the roots of *T. ramosissima* were up-regulated during 48h and 168h under NaCl stress. Note: $p \geq 0.05$ is not marked; $0.01 < p < 0.05$ is marked as *; $0.001 < p < 0.01$ is marked as **; $p \leq 0.001$ is marked as ***.)

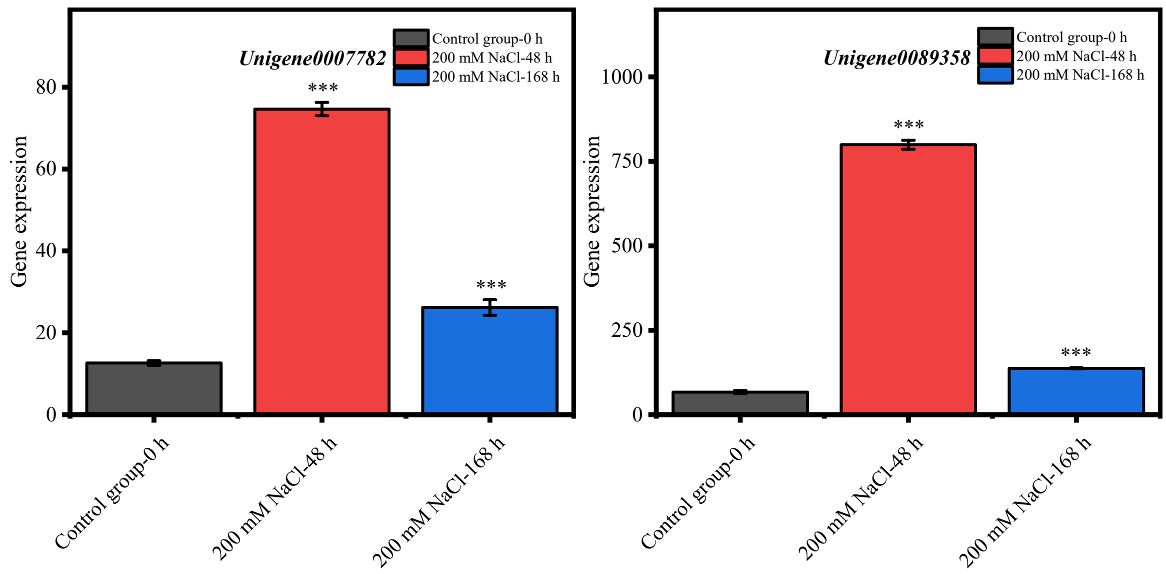

Supplementary Figure S3. The expression levels of candidate key genes in flavonoid biosynthesis pathway (*Unigene0007782* and *Unigene0089358* play an important role in flavonoid biosynthesis pathway of *T. ramosissima* roots at 48h and 168h under NaCl stress. Note: $p \geq 0.05$ is not marked; $0.01 < p < 0.05$ is marked as *; $0.001 < p < 0.01$ is marked as **; $p \leq 0.001$ is marked as ***).

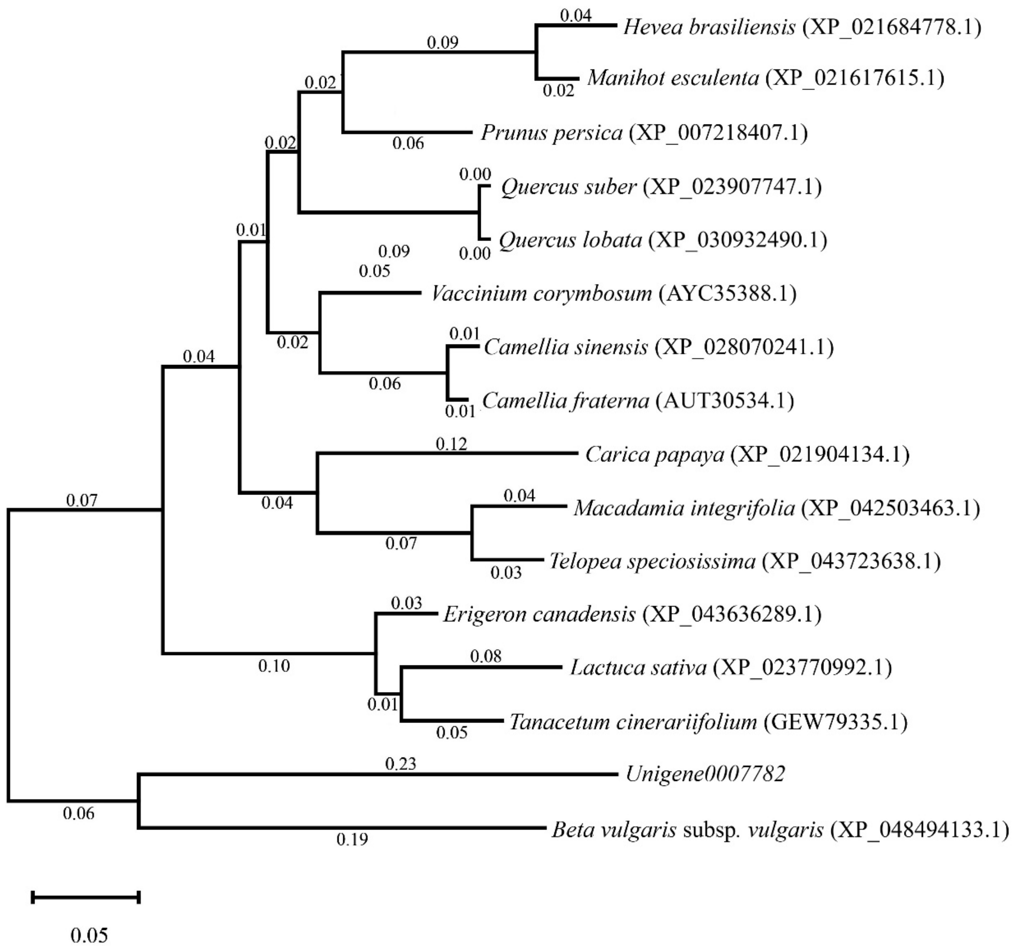

Supplementary Figure S4. Phylogenetic tree analysis of *T. ramosissima* chalcone-flavonone isomerase and other species chalcone-flavonone isomerase (Phylogenetic tree analysis of Unigene0007782 protein amino acid sequence of *T. ramosissima* roots and protein amino acid sequence of other 15 species under NaCl stress).

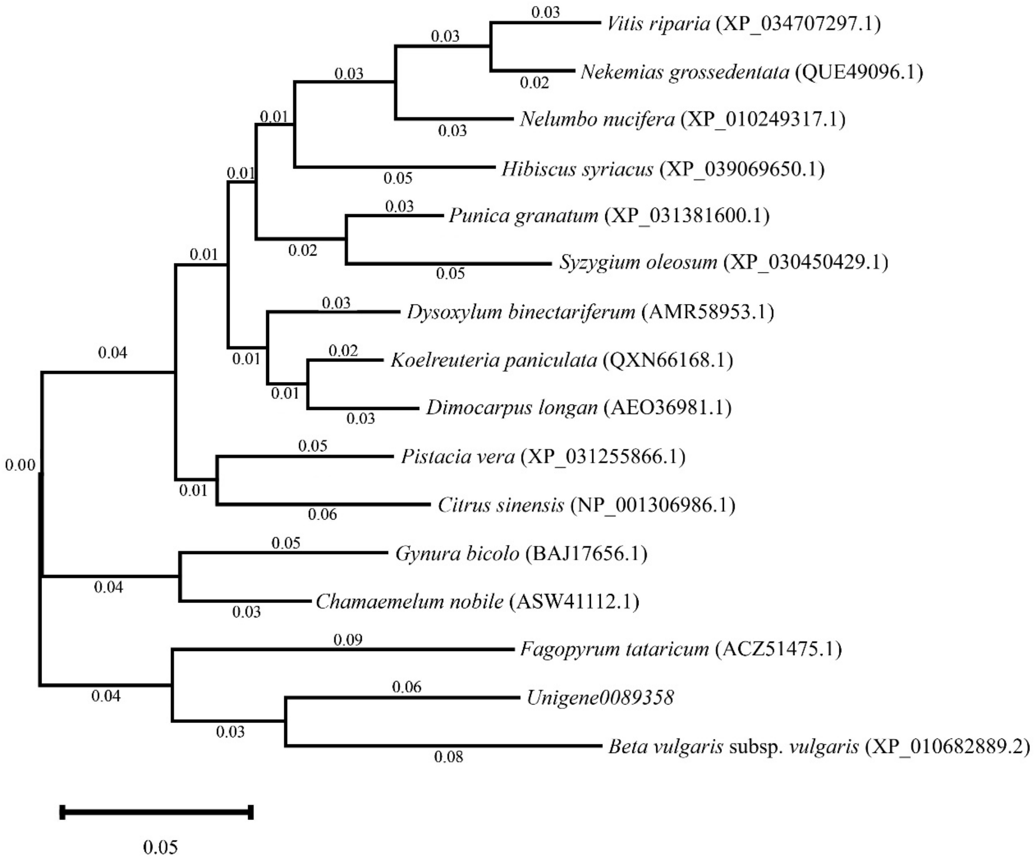

Supplementary Figure S5. Phylogenetic tree analysis of *T. ramosissima* chalcone synthase and other species chalcone synthase (Phylogenetic tree analysis of Unigene0089358 protein amino acid sequence of *T. ramosissima* roots and protein amino acid sequence of other 15 species under NaCl stress).
